# Treatment of chronic insomnia with atypical antipsychotics: results from a follow-up study

**DOI:** 10.5935/1984-0063.20190149

**Published:** 2021

**Authors:** Behnam Khaledi-Paveh, Soroush Maazinezhad, Leeba Rezaie, Habibolah Khazaie

**Affiliations:** 1 Sleep disorders research center, Kermanshah University of Medical Sciences, Kermanshah, Iran.; 2 School of Nursing and Midwifery, Kermanshah University of Medical Sciences, Kermanshah, Iran.

**Keywords:** Chronic Insomnia, Sleep Quality, Atypical Antipsychotics

## Abstract

**Objective:**

Second generation (atypical) antipsychotics are increasingly being used for treatment of insomnia, but there is little evidence to show long-term efficacy of these medication. This follow-up study was designed to assess patients with chronic insomnia who were treated with atypical antipsychotics.

**Material and Methods:**

In this follow-up study, forty patients with chronic insomnia were evaluated between 2016 and 2018 following after one year of treatment with two atypical antipsychotic drugs of olanzapine and quetiapine in two groups in the sleep disorders research center of Kermanshah University of Medical Science in Iran. The Pittsburgh Sleep Quality Questionnaire (PSQI) and 5 consecutive nights of Actigraphy were utilized to evaluate both subjective and objective measures of sleep quality. Lastly, sleep quality before and after treatment and comparisons of sleep quality between the two groups were performed.

**Results:**

Nine male participants comprised olanzapine group (n=22) and six male participants comprised the quetiapine group (n=18). The average age in the olanzapine group was 45.23±10.18 and the average age in the quetiapine group was 46.33±7.99. Results showed total PSQI score improved signiﬁcantly in both groups (p<0.05), while the actigraphy results showed only signiﬁcant improvement in sleep quality parameters in the quetiapine group (p<0.05).

**Conclusion:**

Two atypical antipsychotics drugs of olanzapine and quetiapine have long-term efficacy in managing chronic insomnia. More improvements in objective sleep quality with quetiapine is promising for patients with chronic insomnia. Further research to evaluate long-term adverse effects of atypical antipsychotic drugs is recommended.

## INTRODUCTION

Insomnia is one of the most common sleep disorders in the general population and it is reported by 10-30 percent of the general population. This disorder is typically diagnosed using a subjective complaint about the inability to fall asleep and maintain sleep, along with not having restorative sleep, while experiencing daily consequences such as fatigue, drowsiness, and mood and cognitive symptoms^1^. Between 33 to 50% of adults experience insomnia temporarily from several days to several weeks, however, it is estimated that 20-22% of these individuals experience chronic insomnia^2^. Chronic insomnia, symptoms typically last for over take more than three months, and have the potential to develop cognitive consequences (e.g., problems with memory, attention, concentration and confusion) as well as anxiety, depression, higher risk of committing suicide, drug abuse, and an increase in the risk of cardiovascular diseases and immune deficiency^3^. Additionally, daily drowsiness due to the insomnia increases the risk of motor vehicle accidents and workplace accidents^4^. Therefore, insomnia is considered as a significant health problem and its treatment is of utmost societal.

There are several evidence-based treatments for insomnia including pharmacological treatments and non-pharmacological (such as cognitive-behavioral)^5^ either used in conjunction with one another or separately. Benzodiazepines are among the most common medications for treating insomnia, although concerns for use include increased tolerance levels, potential dependence on the drug, physical side effects such as falls or cognitive impairment in older patients, and hypoventilation in patients with respiratory conditions (including sleep apnea and obesity)^6^ have led to physicians exhibiting increased levels of caution when prescribing these medications and increasingly prescribing sedating antidepressants, particularly trazodone^7^. In a systematic review by Yi et al.^8^, these authors reported that trazodone is effective in sleep maintenance and perceived sleep quality. However, they reported no significant improvement in sleep efficiency and other objective measures. Despite the paucity of information about the potential harmful side effects of trazodone, many physicians prescribe it based on the perception that it is a “safer” sleep-prompting agent compared to alternatives^9^.

Second generation of antipsychotic drugs or atypical antipsychotics are among other medicines that are typically prescribed by the physicians. Although these medicines are generally prescribed for treating psychotic or bipolar disorders, their sedative effects have led them to be used as hypnotics’ drugs in low dosages^10^. Olanzapine and quetiapine are two atypical antipsychotic medications when prescribed in treating insomnia and improvements in sleep quality has been reported following use^11,12^. However, there are a number of factors to be considered when determining whether or not these medications warrant use as first-line treatment of insomnia. Specifically, the studies carried out on these medications are not of high quality, concerns have been reported regarding their long-term use, particularly when considering the increased risk of developing obstructive sleep apnea disorder (OSA), daytime somnolence, extra pyramidal symptoms, or metabolic adverse effects. Therefore, it is not recommended that these medications be prescribed as the first-line treatment for patients with insomnia. That being said, it should be noted that these medications are often prescribed to patients with a history of failure in other treatments or when diagnosed with a comorbid condition^10^. Therefore, conducting additional studies focused on the effectiveness of these medications in treating insomnia could be particularly useful in providing physicians with relevant evidence in support of the best practice for medication use. Considering the fact that the studies conducted on the effectiveness of antipsychotic medicines in treating insomnia are limited in Iran^5^, this study was carried out to follow-up the patients diagnosed with chronic insomnia treated by olanzapine and quetiapine to further examine medication effectiveness within this population.

## MATERIAL AND METHODS

### Study design and setting

The present study is a follow-up study aimed to assess patients diagnosed with chronic insomnia who were treated using olanzapine and quetiapine. This study was conducted in the Sleep Disorders Research Center of Kermanshah University of Medical Sciences, which is the only specialized center for studying and treating sleep disorders in western Iran. Patients treated at this center first receive thorough assessment before receiving pharmacological and non- pharmacological treatment based on protocols and guidelines used for treatment of patients with insomnia^9^. These guidelines include the recommendation that a low dose of atypical antipsychotic be used in cases where there is failure in other treatment.

### Participants

The participants in this study included all the patients diagnosed with chronic insomnia admitted at sleep disorders research center of Kermanshah University of medical sciences (n=55) between 2016-2018. The diagnosis of insomnia was based on the clinical interview carried out by the sleep medicine specialist and International Classification of Sleep Disorders - Third Edition (ICSD-3) diagnostic criteria. Patients were admitted due to complaints of insomnia and reports that common pharmacological insomnia treatments such as benzodiazepines and non-pharmacological treatment had not been effective. Patients diagnosed with psychiatric disorders, substance abuse, and chronic medical disorders were excluded from the present study.

### Measures and study procedures

The present study was approved by the Ethics Committee of Kermanshah University of Medical Sciences and researchers coordinated with a sleep specialist at this facility.

**Figure 1:**
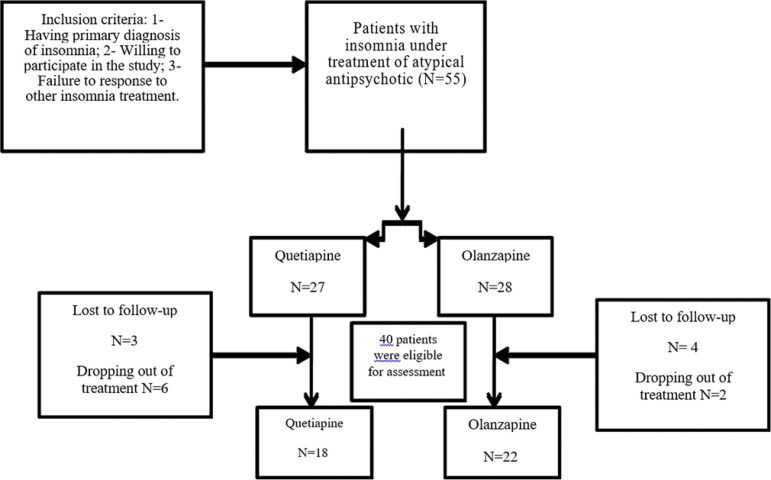
Flowchart of the study.

Recruitment involved one of the research team members who was stationed at the Research Center for Sleep Disorders to choose the eligible patients based on the inclusion/exclusion criteria outlined in the previous section and invite the patients to participate in the study. Following an explanation of the study’s objectives and receiving consent from the patients, each participant filled out the Pittsburgh Sleep Quality Index (PSQI) and actigraphy was administered for five days before beginning treatment. Patients were prescribed one of two medicines of olanzapine (n=28) and quetiapine (n=27) by the sleep specialist. It should be noted that the present study’s design as a follow-up does not warrant the use of random assignment and a placebo group for the prescription of these two drugs, and it was based on the clinical decision of the physician who assess the patients’ general condition. The prescribed doses of olanzapine and quetiapine were 5 and 25mg per night, respectively. The sleep specialist monthly visited patients. After one year, 6 patients dropped out of the olanzapine group and 9 patients dropped out of the quetiapine group. These patients were dropped out of the study are divided into two groups. The first group was comprised of the patients who unilaterally discontinued their treatment and did not respond to any contact from the research team. The second group was comprised of the patients who did continue their treatment and respond to the research team’s contact; however, they stated that they did not want to participate in the follow-up assessment. Forty patients were invited to assess their sleep quality by the research team and asked to fill out the Pittsburgh Sleep Quality Index (PSQI) and actigraphy was administered for five days. Figure 1 shows the flowchart of the present study.

Regarding the subjective assessment of sleep quality, the PSQI was utilized in the present study. This index is a standard questionnaire with validity and reliability demonstrated to be satisfactory when used in Iran^13^. This measure assesses sleep in the past month in seven areas of subjective sleep quality (e.g., sleep latency, sleep duration, habitual sleep efficiency, sleep disturbances, use of sleep medication, and daytime dysfunction). The components of the questionnaire are scored using a Likert scale between 0 and 3 with the lower the scores indicating less dysfunction. The general score of the PSQI is the sum of the seven components that are scored between 0 and 21, with a cut off score of 5 with scores higher than 5 showing poorer sleep quality.

Objective assessment of sleep quality included actigraphy lasting five days. Actigraphs’ are portable devices, typically the size of a wristwatch that can be given to research centers in order to monitor the sleep quality of patients. The present study monitored sleep quality for a duration of five days. The parameters that are recorded by actigraphy include total sleep time (TST), sleep efficiency (SE), sleep latency (SL), wake up after sleep onset (WASO)^14^. Ambulatory monitoring act watch was utilized in the present study. These two assessments were administered to participants before treatment began and a year after treatment was completed.

### Data analysis

Descriptive analysis was used for the demographic properties. In order to study the intergroup changes, the Wilcoxon test and paired t and to compare the means between the two groups Mann-Whitney test and independent t were used. The significance level for all the tests was considered to be lower than 0.05. The collected data was analyzed using SPSS v. 21.

### Ethical approval

The present study was approved by the Ethical Committee of Kermanshah University of Medical Sciences (IR.KUMS.REC.1397.006). All procedures performed in studies involving human participants were in accordance with the ethical standards of the committee and based on the 1964 Helsinki declaration and its later amendments or comparable ethical standards. Informed consent was obtained from all participants included in the study.

## RESULTS

Nine male participants were in the olanzapine group and six male participants were in the quetiapine group. The average age in the olanzapine group was 45.23±10.18 and the average age in the quetiapine group was 46.33±7.99. The BMI of both group members was higher than 25 and all members were overweight. Other demographic properties are presented in (Table 1).

**Table 1:** Characteristics of patients participating in the study.

		Group					
		Quetiapine			Olanzapine		
		Count	Column N %	Mean±SD	Count	Column N %	Mean±SD
Gender	MALE	6	33.3%		9	40.9%	
	FEMALE	12	66.7%		13	59.1%	
AGE				46.33±7.99			45.23±10.18
Marital status	Married	15	83.3%		18	81.8%	
	Single	3	16.7%		4	18.2%	
Education	illiterate	5	27.8%		5	22.7%	
	Under the diploma	4	22.2%		8	36.4%	
	diploma	5	27.8%		2	9.1%	
	Academic	4	22.2%		7	31.8%	
BMI				28.11± 5			27.07± 3.25
Job status	Employee	6	33.3%		9	40.9%	
	free job	2	11.1%		4	18.2%	
	Unemplo yed	10	55.6%		9	40.9%	

The results for the subjective portion of study PSQI showed that the Pittsburg Sleep Quality General Index had a significant improvement in both groups (*p*<0.05). Results suggested that the determining parameters of sleep quality in Pittsburg index, except for the parameters of use of sleep medication, and daytime dysfunction, had a significant improvement in the quetiapine group (*p*<0.05).

Results from the objective assessment of sleep quality using the actigraph in the quetiapine group showed an increase in the TST average (*p*=0.006), an increase in SE average (*p*=0.002), and a decrease in the WASO average (*p*<0.001). The changes in TST, SE and SL averages were not significant in the olanzapine group (*p*<0.05). However, the WASO average had a significant decrease (*p*<0.001). The intergroup results comparison on TST parameter in the two olanzapine and quetiapine groups showed that a significant difference in TST mean was present after a year (*p*=0.049). Table 2 presents the intragroup and intergroup changes in subjective and objective sleep quality parameters parameter in the two olanzapine and quetiapine groups.

**Table 2:** Changes in mean components of Pittsburgh questionnaire and actigraphic indices before and after one year in both olanzapine and quetiapine groups

	Variables	Quetiapine Group		*P*	Olanzapine Group		*P*	*P*
		Baseline Mean± SD	Outcome Mean± SD	Within the group	Baseline Mean± SD	Outcome Mean± SD	Within the group	Between the two groups after treatment
PSQI	SSQ	2.33±.76	1.22±.73	<0.001*	2.27±.82	1.63±.84	0.005*	0.112***
	SL	2.66±.48	2±.68	0.001*	2.45±.59	1.95±.65	0.005*	0.861***
	SDU	2.50±.70	1.94±.80	0.013*	2.31±.71	1.68±.77	0.001*	0.381***
	SE	2.66±.84	2.11±1.02	0.031*	2.45±.91	1.63±1.13	0.009*	0.209***
	SDB	1.66±.68	1.16±.38	0.013*	1.63±.58	1.40±.50	0.166*	0.199***
	USM	2.16±1.24	2.16±1.20	0.100*	1.86±1.24	1.81±1.00	0.836*	0.240***
	DD	1.66±.90	1.27±.57	0.134*	1.72±.93	1.22±.75	0.008*	0.619***
	TPS	15.94±2.89	11.77±2.43	<0.001*	14.77±2.87	11.36±3.06	0.001*	0.757***
Actigraphy	TST	339.78±91.51	416.30±48.58	0.006**	373.31±68.94	381.73±56.96	0.592**	0.049****
	SE	77.49±19.22	92.53±6.84	0.002*	78.79±13.14	83.60±9.13	0.149*	0.209***
	SL	27.26±37.83	24.02±27.27	0.679*	25.43±17.22	27.18±14.33	0.958*	0.045***
	WASO	11.72±14.84	2±2.47	<0.001*	7.27±4.47	1.54±1.53	<0.001*	0.778***

Data were presented as mean ± standard deviation:

* Wilcoxon Signed Ranks Test;

** Paired t test;

*** Mann-Whitney U;

**** Independent T; **SSQ:** Subjective sleep quality; **SL:** Sleep latency; **SDU:** Sleep duration; **SE:** Sleep efficiency; **SDB:** Sleep disturbance; **USM:** Use of sleep medication; **DD:** Daytime dysfunction; **TPS:** Total PSQI Score; **TST:** Total sleep time; **WASO:** Wake up after onset sleep.

## DISCUSSION

As the first study conducted on the follow-up of the patients diagnosed with chronic insomnia treated by two new generation antipsychotic medicines quetiapine and olanzapine in Iran, this study showed that there is a significant difference in the sleep quality of the patients, comparing before and after treatment (*p*<0.05). These findings suggest the general satisfaction when prescribed these medicines between both groups. Objective assessment of sleep using 5 days of actigraphy showed significant changes in actigraphy indices (TST, SE and WASO) in the quetiapine group (*p*<0.05). However, significant changes were observed in olanzapine group only in the WASO index. Considering the significant decrease of sleep disturbances WASO in both groups, the subjective satisfaction both groups of patients regarding on their night sleep could be attributed to this. The positive influence of quetiapine on the sleep quality in patients diagnosed with Parkinson, Alzheimer’s and the positive influence of olanzapine on the sleep quality in patients diagnosed with insomnia are have been reported in the previous studies^5,15-17^. Positive influence of these two medicines on the examined sleep variables should be seen in light of the affinity of atypical antipsychotics agents to central nervous system receptors 5-HT2A/2C-antagonist. Central occupancy of the receptor, primarily 5-HT2C, and so the blockage of H1 receptors and alpha-1adrenoreceptors has also been proposed as a possible mechanism underlying some of the hypnotic effects of these medications^18^.

Comparing the effectiveness of these two medications indicated that significant positive changes in actigraphy indices are observed more in the patients treated by quetiapine. In 2007, Giménez et al.^19^ explained that olanzapine could be effective in improving the objective indices of total sleep time and sleep efficiency, while in this study, significant positive changes were observed in the group treated by quetiapine. In 2005, Juri et al.^15^ stated that a 31mg dosage of quetiapine per night could be a safe medicine in improving insomnia. In a study on the effect of quetiapine on primary insomnia, Wiegand et al.^10^ stated that after 6 weeks of treatment by 75mg doses per night, significant changes were observed in the objective indices of TST and SE, and the Pittsburg index in subjective assessment, with non-significant changes identified in SL. These results supports the findings of the present study with the only difference being that participants in this study were prescribed quetiapine with lower doses of 25mg per night for a year. Thompson et al.^11^ stated that quetiapine should not be prescribed as the first line of treatment in primary insomnia, while the patients in this study were in support of treatment using second generation antipsychotic medicines after considerable time following the onset of insomnia and previously failed treatment attempts. Participants in the olanzapine group reported being satisfied regarding sleep quality on the Pittsburgh Sleep Quality Index, despite the increase in their night sleep duration in actigraphy. This finding can be attributed to the significant decrease in the average sleep disturbances in this group. Khazai et al^20^ study suggests that creating a sense of acceptance and satisfaction toward treatment in the minds of the patients diagnosed with chronic insomnia could have a significant role in the adjustment of their emotions and subsequently their sleep quality due to improvement in treatment adherence. Results suggested that the BMI of these individuals was in the overweight range. This is an important finding due to BMI’s relationship with the risk of OSA occurrence, although there is evidence that there is an identical level of risk of developing of OSA from the consumption of these medications without an increase in weight^5^. Therefore, assessing the risk of OSA in the patients who take these medications critical when physicians consider this treatment.

## CONCLUSION

Despite chronic insomnia typically having adverse effects on quality of life, many challenges are present regarding the treatment and management of this condition. In many cases, common treatments do not lead to desirable results. The findings of this study suggest that prescribing second generation antipsychotic medicines such as quetiapine and olanzapine could be effective in improving sleep quality in these patients. Therefore, it is recommended that the prescription of these medications be considered after examining a patient’s specific condition, circumstances, and risk, with special attentions given to use of these medications by sleep specialists in treatment programs. It is also recommended that future studies further assess potential side effects in both the short- and long-term.

### Strengths and limitations

This study that is the first of its kind in Iran, which comes with specific strengths and limitations, should be acknowledged. Regarding the first strength of this study, the use of both subjective and objective assessments of sleep quality lasting five days provided the researchers with more accurate information compared in relying solely on a singular assessment method. The one-year follow-up ensured that patients demonstrated treatment efficacy using the medication with constant doses without developing tolerance in the long-term, which could be used for planning the treatment in these patients. However, the study also has some limitations that should be acknowledged. First, the lack of subjective and objective sleep quality assessments in shorter three-month and six-month intervals is the main limitations of this study. Second, polysomnography indices could provide more complete information on the sleep structure changes; however, these were not available in this study due to the high costs of polysomnography. Finally, since the main objective of this study was not to compare the effectiveness of olanzapine and quetiapine, the side-effect assessment of these medications was limited to the individual doctors’ visits and no official assessment was carried out by the researchers. Future studies that take into account these limitations, as well as increase the sample size, is recommended.
